# Role of estrogen receptor signaling pathway-related genes in diffuse large B-cell lymphoma and identification of key targets *via* integrated bioinformatics analysis and experimental validation

**DOI:** 10.3389/fonc.2022.1029998

**Published:** 2022-11-29

**Authors:** Bo Chen, Tianjiao Mao, Xiuni Qin, Wenqi Zhang, Nobumoto Watanabe, Jiang Li

**Affiliations:** ^1^ Guangdong Engineering Research Center of Oral Restoration and Reconstruction, Affiliated Stomatology Hospital of Guangzhou Medical University, Guangzhou, Guangdong, China; ^2^ Guangzhou Concord Cancer Center, Guangzhou, Guangdong, China; ^3^ School of Basic Medicine, Guangzhou Medical University, Guangzhou, Guangdong, China; ^4^ Chemical Biology Research Group, RIKEN Center for Sustainable Resource Science, Wako, Saitama, Japan; ^5^ Bio-Active Compounds Discovery Unit, RIKEN Center for Sustainable Resource Science, Wako, Saitama, Japan

**Keywords:** diffuse large B-cell lymphoma (DLBCL), estrogen receptor (ER), biomarker, bioinformatics, prognosis

## Abstract

Diffuse large B-cell lymphoma (DLBCL) is a highly heterogeneous malignancy. Epidemiologically, the incidence of DLBCL is higher in men, and the female sex is a favorable prognostic factor, which can be explained by estrogen. This study aimed to explore the potential targets of the estrogen receptor (ER) signaling pathway and provide a meaningful way to treat DLBCL patients. Datasets were obtained from the Gene Expression Omnibus (GEO) to identify differentially expressed genes (DEGs). Representative gene sets estrogen receptor pathways, and growth regulatory pathways were identified based on Gene Set Enrichment Analysis (GSEA) analysis. Gene Ontology (GO) and Kyoto Encyclopedia of Genes and Genomes (KEGG) were used for function and pathway analysis. STRING and Cytoscape were used to construct the interaction network, and the MCODE plug-in performed the module analysis. GEPIA, TCGA, and LOGpc databases were used for expression and predictive analysis. The Human Protein Atlas (HPA) database was used to analyze the protein expression levels, cBioPortal was used to explore genetic alterations, and ROC analysis and prognostic assessment were used to predict the diagnostic value of genes. Finally, BJAB cells were treated with ER inhibitor fulvestrant and specific shRNA, and the expression of hub genes was verified by RT-qPCR. We identified 81 overlapping DEGs and CDC6, CDC20, KIF20A, STIL, and TOP2A as novel biomarkers affecting the prognosis of DLBCL. In addition, the STAT and KRAS pathways are considered potential growth regulatory pathways. These results hold promise for new avenues for the treatment of DLBCL patients.

## Introduction

Diffuse large B-cell lymphoma (DLBCL) is a highly heterogeneous tumor and is the most common subtype of aggressive non-Hodgkin lymphoma (NHL) in adults, accounting for approximately 40% (1–3). Men are more affected than women, with a median ratio of about 1.4 (4). Gene expression profiling revealed that the disease is divided into three subtypes: germinal center B-cell-like (GCB), activated B-cell-like (ABC), and primary mediastinal B-cell lymphoma (5, 6). DLBCL often presents as aggressive lymphomas, particularly ABC-DLBCL, characterized by high activation of nuclear factor kappa B (NF-κB) signaling (7, 8). Although patient outcomes have improved by combining CHOP chemotherapy (cyclophosphamide, doxorubicin, vincristine, and prednisolone) with the monoclonal anti-CD20 antibody rituximab (9, 10), however, 30-40% of patients still develop drug resistance during treatment (11–15). The application of next-generation sequencing has revealed a significant degree of molecular and clinical heterogeneity in DLBCL (16–18). This heterogeneity presents a series of challenges to the understanding and treatment of DLBCL. Further deciphering the genes and signaling pathways involved in the initiation and progression may provide opportunities for effective treatments of DLBCL.

Epidemiological studies have shown that the incidence and prognosis of women are significantly better than those of men in DLBCL (19–21). In addition to higher morbidity, male patients also showed worse outcomes (22). A population-based study found that women younger than 52 years had better overall survival compared with men of the same age in DLBCL. This difference disappeared in women after menopause, suggesting an effect of estrogen (23). Pregnancy has been reported to reduce the incidence of NHL, suggesting that female sex hormones are involved in the development of NHL (24, 25). The study showed that when T-cell lymphomas were transplanted into mice, male mice developed larger tumors than female mice, which were eliminated after oophorectomy (26). In addition, the human Burkitt lymphoma cell line Raji or the Mantle lymphoma cell line Granta-519, treated with estradiol or a selective ERβ agonist, inhibited lymphoma growth (27). All the above results suggest that estrogen signaling plays an essential regulatory role in the occurrence and development of lymphoma.

The cellular action of estrogen is mediated through the estrogen receptor (ER), which is divided into two forms, ERα and ERβ (28, 29). Among them, ERβ is abundantly present in normal B lymphocytes, while ERα is present in other tissues, such as reproductive organs (29). Studies have shown that ERα confers growth and proliferation signals while ERβ mediates anti-proliferative and pro-apoptotic effects on lymphocytes (30). Selective ERβ receptor agonists (KB9520) have been developed, and these compounds have therapeutic potential as growth regulators for lymphoid malignancies (26). Therefore, the targeted development of new targets related to ER signaling is a practical means for treating lymphoma patients, including DLBCL.

Our study downloaded the DLBCL datasets GSE56315 and GSE25638 from the Gene Expression Omnibus (GEO) database. Differentially expressed genes (DEGs) were screened by comparing gene expression between DLBCL and normal samples. Based on the GSE56315 dataset, we performed Gene Set Enrichment Analysis (GSEA) analysis to identify representative gene sets, including estrogen receptor and growth regulatory pathways. STRING and Cytoscape were used to construct protein-protein interaction (PPI) networks, and modular analysis was performed, identifying five hub genes (CDC6, CDC20, KIF20A, STIL, and TOP2A). Then, enrichment analysis was performed using Gene Ontology (GO) and Kyoto Encyclopedia of Genes and Genomes (KEGG) terms from the DAVID database, explaining the critical functions and pathways involved in DEGs. The expression of hub genes in DLBCL was analyzed by GEPIA and TCGA databases, Receiver Operator Characteristic Curve (ROC) analysis and prognostic assessment were performed to predict the diagnostic value of biomarkers, and the LOGpc database was used to analyze the survival of genes in DLBCL. In addition, we obtained hub gene immunohistochemistry results from the Human Protein Atlas (HPA) database and performed a genetic alteration analysis of the genes using the cBioPortal database. Finally, to verify the expression of the hub genes, we treated the BJAB cells with the ER inhibitor fulvestrant and specific shRNA, respectively, and applied RT-qPCR technology to detect the expression of the hub genes.

In conclusion, this study explores a promising new target molecule related to the ER pathway that predicts the prognosis of DLBCL and analyzes that the STAT and KRAS pathways may be the potential growth regulation pathways of DLBCL. Targeting CDC6, CDC20, KIF20A, STIL, and TOP2A may represent a new strategy for therapeutic intervention in DLBCL.

## Materials and methods

### Acquisition of microarray datasets

The DLBCL datasets (GSE56315 and GSE25638) were obtained from the GEO (http://www.ncbi.nlm.nih.gov/geo/) database. GSE56315 contains 55 DLBCL and 33 normal samples (31); GSE25638 contains 26 DLBCL and 13 normal samples (32). Both microarray datasets are based on the GPL570 platform (Affymetrix Human Genome U133 Plus 2.0 Array).

### Identification of differentially expressed genes

The robust multi-array (RMA) mean algorithm in the “Affy” package in R language (http://cran.r-project.org/) was applied to raw data for high-throughput functional genome expression, including background correction, normalization, and probe summary. Statistically significant DEGs were mined based on the difference in expression values between DLBCL and normal samples using linear models from the “LIMMA” package in the R language. Volcano plots of DEGs were generated using the “ggplot2” package in the R language, and Benjamini-Hochberg’s method was used to control for the false discovery rate (FDR). P-value < 0.05 and | log2FC | > 1 were critical values for screening DEGs.

### Gene and functional set enrichment analysis

GSEA analysis with MSigDB provided by the JAVA program was applied to explore the biological processes influenced by gene expression in the DLBCL dataset. 22,161 genes were included in the GSEA analysis process. The Hallmark gene set was used in this study. Gene sets that obtained the highest enrichment score (ES) with normalized P-value < 0.05 and FDR < 0.25 were considered significantly enriched.

### PPI network construction and module analysis

STRING (v 11.5, https://string-db.org) is an online tool for retrieving interacting genes and proteins, entering genes into databases to construct PPI networks that show physical and functional interactions. This study selected proteins with a total score > 0.4 for network construction. In addition, Cytoscape (v 3.8.2, https://cytoscape.org) was used to visualize the network, the Network Analyzer calculated the node degree, and the network was plotted with different colors and sizes showing the modulation and node degree. Finally, the key modules in the network are identified by the MCODE plug-in for screening hub genes.

### Validation of hub gene expression at mRNA and protein expression levels

The mRNA expression of genes was explored by gene expression profiling analysis (GEPIA; http://gepia.cancer-pku.cn) and the TCGA database. Among them, the GEPIA database includes 46 DLBCL and 337 normal samples, and the TCGA database includes 47 DLBCL and 444 normal samples. The criteria for selecting the reducer were as follows: |log2FC| > 1, P-value < 0.05. In addition, immunohistochemical data from the Human Protein Atlas (HPA) database (https://www.proteinatlas.org) were used to analyze the protein expression levels of the genes.

### Genetic alterations of hub genes

The cBio Cancer Genomics Portal (http://www.cbioportal.org/) is an open platform that provides visualization, analysis, and download of large-scale cancer genomic datasets across various cancer types. Complex cancer genome maps are readily available using the portal’s query interface, allowing researchers to explore and compare genetic changes between samples. The cBioPortal explores genetic alterations in genes, frequency of alterations, and overall survival.

### Biomarker predictive value and prognostic assessment

The “pROC” package performed ROC analysis in R language to predict the diagnostic validity of biomarkers. The area under the ROC curve value (AUC) was used to determine the diagnostic validity of DLBCL versus control samples in the dataset. LOGpc (https://bioinfo.henu.edu.cn/DatabaseList.jsp) is a pan-cancer long-term outcome and gene expression profiling database containing 209 expression datasets, 13 for 31,310 patients with 27 different malignancies survival terms. Here, we screened genes for survival analysis of gene expression in relevant tumors through the LOGpc database, examined the relationship between gene expression and patient prognosis in DLBCL, and generated relevant survival curves.

### Cell culture and handling

#### Cell lines

BJAB cell lines, donated by Professor Xiaoren Zhang from Guangzhou Medical University Affiliated Cancer Hospital/Oncology Institute, State Key Laboratory of Respiratory Diseases, using RPMI 1640 medium containing 10% fetal bovine serum. Cells were cultured in an incubator at 37°C, 5% CO2, and saturated humidity, and passaged for 2-3 days. The experiments used cells in a logarithmic growth phase.

Take the cell suspension containing 1.5 × 10^5^ cells/ml, inoculate it in a 6-well plate (1ml/well), and use RPMI1640 medium without dual antibodies (penicillin and streptomycin). When the cell density reached 50%-60%, the cells were treated with 100nM ER inhibitor fulvestrant (MCE, China) and shRNAs (ERβ-shRNA, vector) (Eurogentec, Belgium), respectively. The target sequences of ERβ-shRNA are as follows: 5’GCTTCAGGC-TACCATTATGttcaagagacataATGGTAGCCTGAAGCttttttac-gcgt-3’; EZ Trans transfection reagent was used for transfection, and the operation was carried out according to the instructions. After 48h, cells were collected to extract RNA, and mRNA levels were detected by RT-qPCR technology.

### Real-time quantitative PCR

Total RNA was extracted using kit reagents (Invitrogen), the amount of RNA was 5µg, and the GoScript™ Reverse Transcription System Kit (Promega, UK) was used. Using SYBR Premix Ex Taq™ II (TaKaRa Clontech), cDNA was reverse transcribed for qPCR following the manufacturer’s instructions. Gene expression analysis was performed using the 2(−ΔΔCt) method. Primers were used to detect the gene of interest (**Table 1**), and all primers were purchased from Sangon Biotech (Shanghai, China). The PCR reaction program was as follows: 95°C for 30 s, 95°C for 5 s, and 60°C for 34 s, for a total of 40 cycles. Analysis was performed using the ΔΔCt method, target gene mRNA = 2-ΔΔCt.

**Table 1:** The primer sequences of qPCR.

Primer Name	Forward primer (5′–3′)	Reverse primer (5′–3′)
ESR2	CCAGGTTCAAAGAGGGATGCTCAC	TCCTTCACACGACCAGACTCCATAG
CDC6	AGGCACAGGCTACAATCAGTTTTCC	CGAGGAGAACAGGTTACGGTTTGG
CDC20	GGAGTGCAAGCTCTGGTGACATC	GGTGCCCACAGCCAAGTAGTTG
KIF20A	GCCGCAGTCACAGCATCTTCTC	TTCCTTCAACCGTTCACCACTCTTC
STIL	GTGACTGAGAAGACCATCCGACTTG	ACCTTCTTCATCTTCGTCTGCTGTC
TOP2A	TACCACTGTCTTCAAGCCCTCCTG	TGCTGCTGTCTTCTTCACTGTCAC
β-actin	ATCACTATTGGCAACGAGCGGTTC	CAGCACTGTGTTGGCATAGAGGTC

### Statistical analysis

Statistical analysis was performed using GraphPad 9. Data are presented as mean ± standard deviation (X ± SD). The data between the two groups were compared using the t-test method. P < 0.05 was considered statistically significant.

## Results

**Figure 1:**
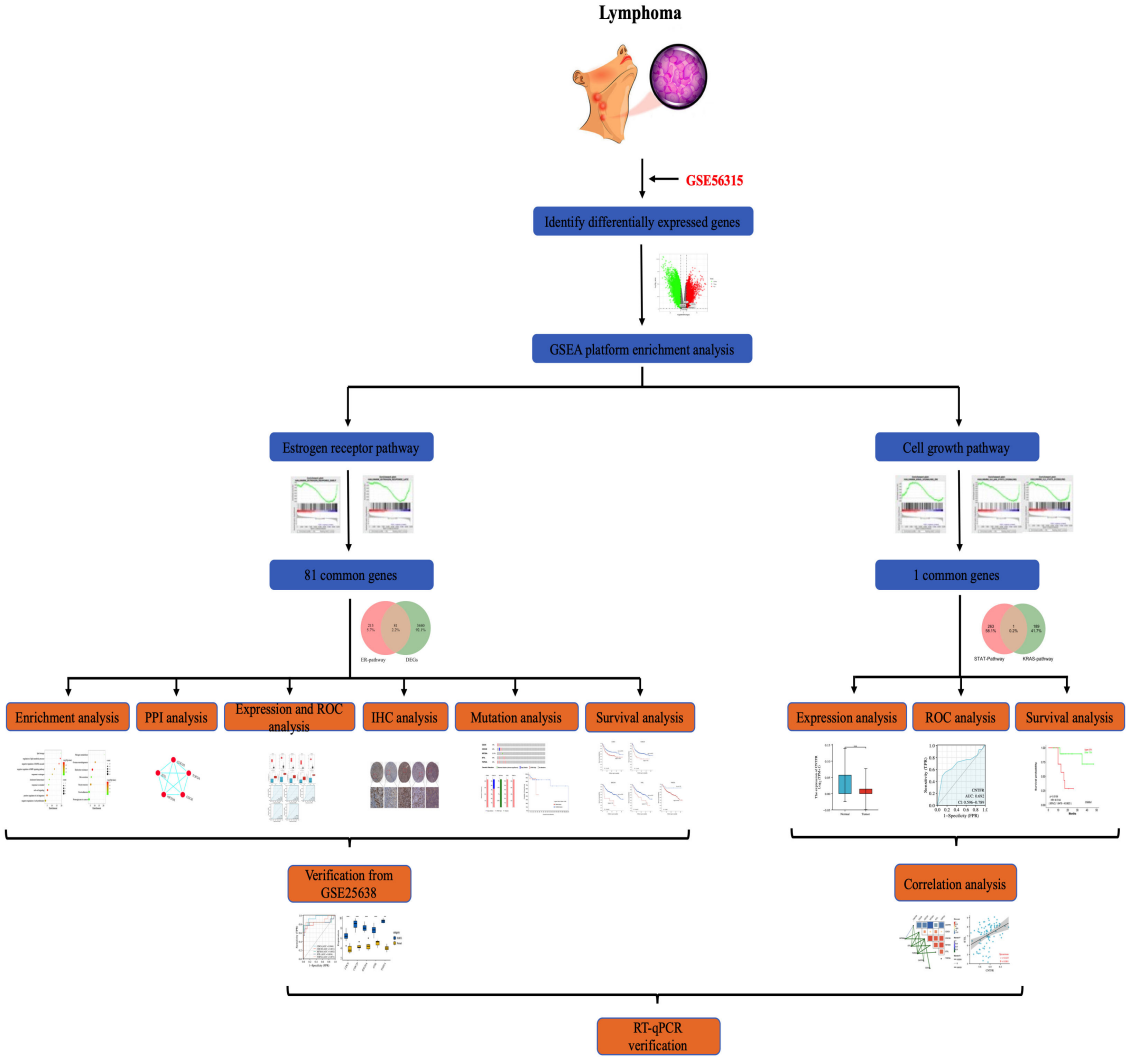
Flowchart showing.

The overall procedure for data collection and analysis followed in this study is illustratively described in **Figure 1**.

### Processing of microarray datasets

**Figure 2:**
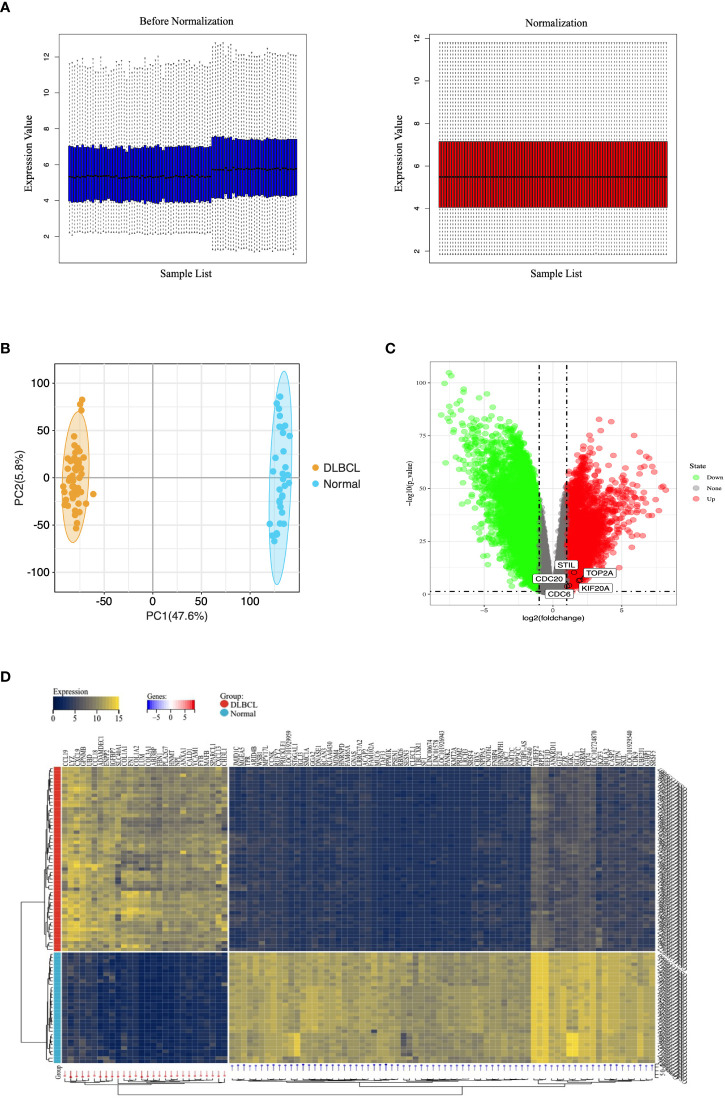
Microarray data of DEGs in the GEO database. **(A)** Normalization of the microarray datasets. The black line in each box represents each data group’s median, which determines the degree of normalization of the data through its distribution. The left side is the expression value data before normalization, the right side is the normalized expression value data. **(B)** PCA diagram of samples based on expression abundance. **(C)** The volcano of DEGs. The green points indicate the screened down-regulated DEGs, the red points indicate the screened up-regulated DEGs, and the gray points indicate genes with no significant differences. **(D)** The heatmap for top 100 DEGs. All DEGs are screened based on P-value < 0.05 and |fold change| > 1. DEGs, differentially expressed genes; PCA, Principal Component Analysis.

To remove systematically biased genes in the raw data, we preprocessed the microarray dataset using the “Affy” package in the R langauge. **Figure 2A** shows gene expression data before and after normalization. The black line in each box represents the median value for each microarray dataset. The black bars appear at nearly the same level in the boxplots, indicating that the normalization effect is significant. Based on the normalized data, we completely differentiated DLBCL from the normal group by PCA analysis (**Figure 2B**). The DEGs were screened using the “limma” package in the R language, with “P-value < 0.05, |log2FC| > 1” as the filter conditions. 9,806 DEGs were obtained, including 3,521 up-regulated genes and 6,285 down-regulated genes. The volcano plot was used to show the differential expression of genes in the dataset (**Figure 2C**), and the heatmap was used to indicate the term of the top 100 (**Figure 2D**).

### The representative gene sets in GSEA analysis

**Figure 3:**
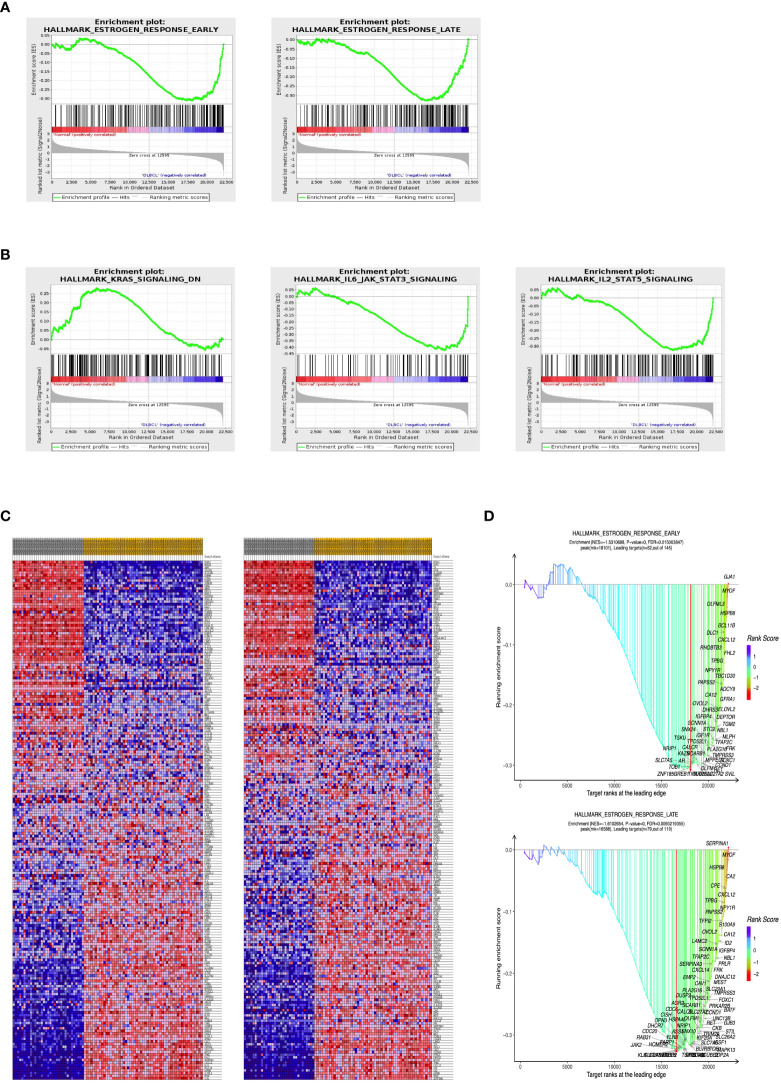
GSEA plot showing most enriched pathways in the DLBCL. GSEA results showed **(A)** estrogen receptor signaling and **(B)** growth regulation signaling pathways were enriched mainly in DLBCL. **(C)** Heatmap distribution of genes involved in ER signaling. **(D)** The distribution of genes is in the GSEA dot plot. ER, estrogen receptor. GSEA, Gene and functional set enrichment analysis; ES, Enrichment score; FDR, false discovery rate; NES, normalized ES.

To identify signaling pathways involved in DLBCL regulation, we performed a GSEA analysis to compare DLBCL and normal groups. This enriched representative gene sets contained estrogen receptor early, estrogen receptor late, KRAS signaling, IL6 JAK STAT3 signaling, and IL2 STAT5 signaling. As shown in **Figures 3A, B**, these pathways may affect DLBCL development by regulating cell growth. Among them, the expression and distribution of ER pathway-related genes are shown in **Figures 3C, D**.

### Identification of overlapping DEGs

**Figure 4:**
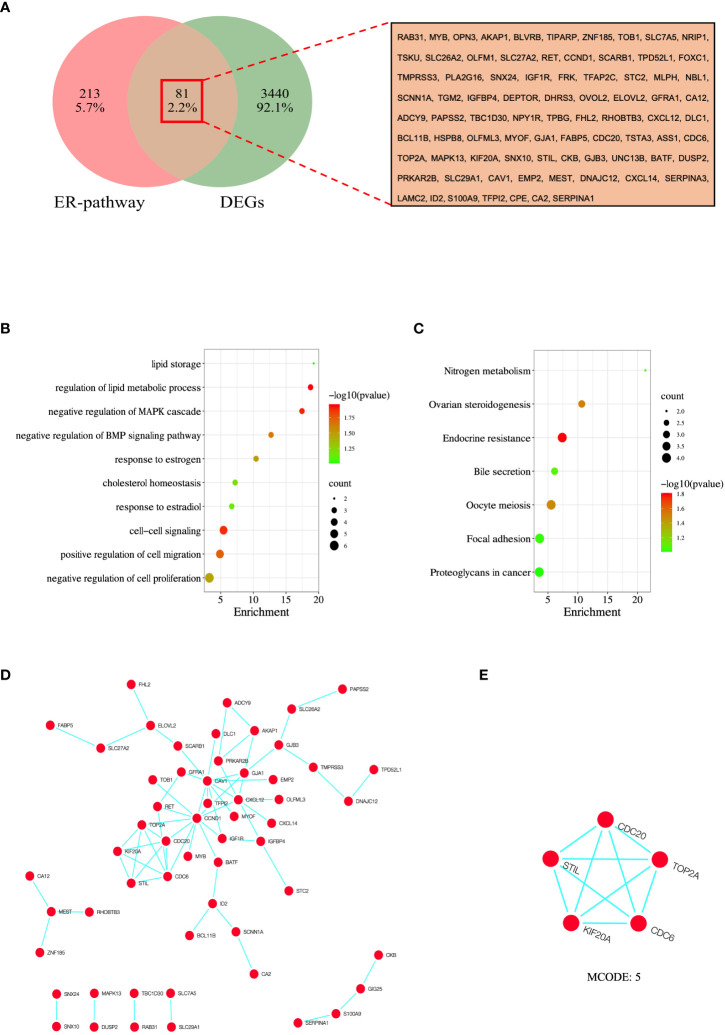
Analysis and screening of hub genes. **(A)** Venn diagram of 81 overlapped DEGs of ER-pathway and DEGs. **(B)** GO enrichment analysis of the overlapped DEGs. **(C)** KEGG enrichment analysis of the the overlapped DEGs. **(D)** PPI network of overlapped DEGs was constructed in Cytoscape, and **(E)** MCODE analysis. GO, Gene Ontology; KEGG, Kyoto Encyclopedia of Genes and Genomes; PPI, protein-protein interaction.

To identify overlapping DEGs, we used the venny online tool to analyze ER pathway-related genes and DEGs and took the overlapping portion of the two datasets. In this study, we identified 81 overlapping DEGs (**Figure 4A**).

### GO and KEGG enrichment analysis of overlapping DEGs

To further understand the molecular functions and pathways involved in overlapping DEGs, we performed enrichment analysis using the DAVID database. The results show that these DEGs are mainly interested in lipid storage, regulation of lipid metabolic process, negative regulation of MAPK cascade, negative regulation of BMP signaling pathway, response to estrogen, cholesterol homeostasis, response to estradiol, cell-cell signaling, positive regulation of cell migration, and negative regulation of cell proliferation were significantly enriched (**Figure 4B**). In addition, KEGG analysis showed that DEGs were significantly enriched in nitrogen metabolism, ovarian steroidogenesis, endocrine resistance, bile secretion, oocyte meiosis, focal adhesion, and proteoglycans in cancer (**Figure 4C**).

### Construction of PPI network and identification of hub genes

To establish protein-protein interactions, we constructed a PPI network for overlapping DEGs using the STRING online database, involving 81 nodes and 67 edges. Non-interacting proteins were filtered out using Cytoscape software and visualized (**Figure 4D**). Finally, through the algorithm of the MCODE plug-in, the module with the highest score was selected as the key module (**Figure 4E**), and the genes in the key module (CDC6, CDC20, KIF20A, STIL, and TOP2A) were selected as the hub genes.

### Expression and predictive value of genes

**Figure 5:**
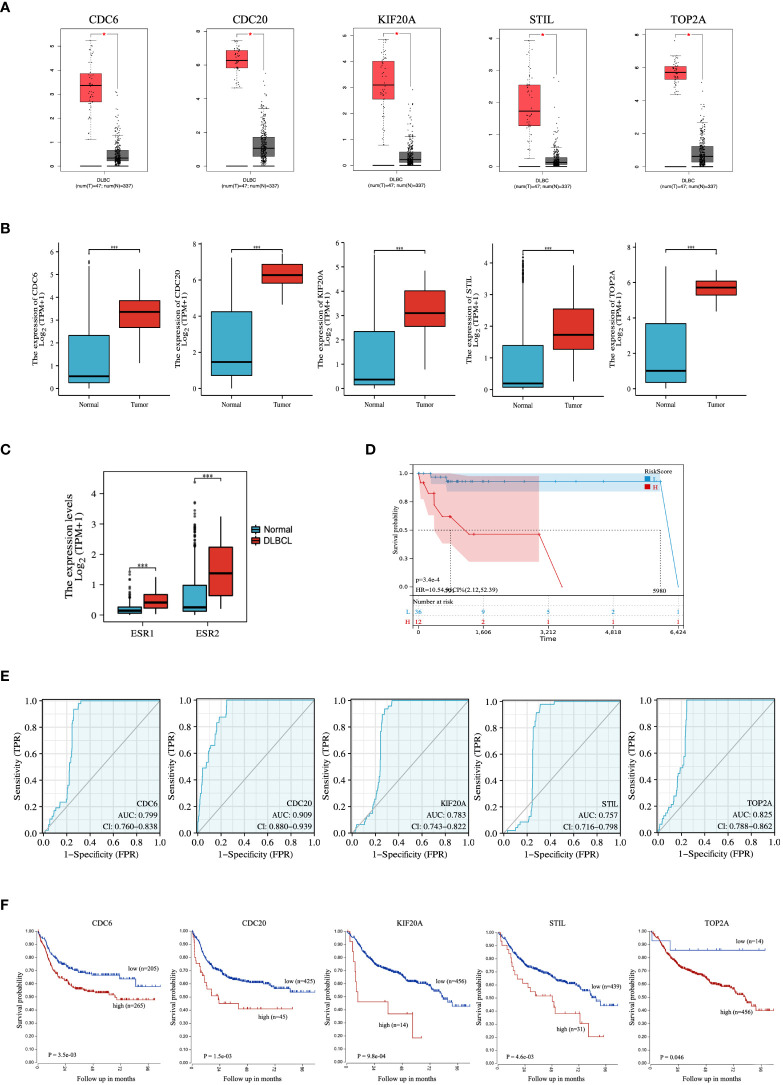
Expression and ROC analysis of hub genes. Expression levels of the hub genes between DLBCL and normal tissues in **(A)** GEPIA and **(B)** TCGA database. **(C)** Expression levels of ER between DLBCL and normal tissues in TCGA database. **(D)** LASSO-cox analysis of ER pathway-related genes. **(E)** ROC analysis and **(F)** prognostic assessment of hub genes. ROC, Receiver Operator Characteristic Curve. *P < 0.05; ***P < 0.001.

The expression of hub genes between DLBCL and normal tissues was investigated using the GEPIA database. The number of samples in the DLBCL group was 47, and the number of samples in the normal group was 337. At the same time, the results were validated using the TCGA database. The results indicated that the mRNA expression of hub genes in the GEPIA database was significantly higher in DLBCL than in the normal group (P < 0.05) (**Figure 5A**), and the mRNA expression of hub genes in the TCGA database was also significantly higher in DLBCL than in the normal group (P < 0.001) (**Figure 5B**). Combined with the analysis results of GEPIA and TCGA databases, the expressions of CDC6, CDC20, KIF20A, STIL, and TOP2A were all higher than those in normal tissues in DLBCL. The expression of ER (ESR1 and ESR2) between DLBCL and normal tissues was further evaluated by the TCGA database, and the results showed that the mRNA expression of ER was significantly higher in DLBCL than normal group (P <0.001) (**Figure 5C**). Using the clinical data of DLBCL patients in the TCGA database, lasso-cox analysis of ER pathway-related genes showed that ER pathway correlates with poor prognosis of the DLBCL patients (**Figure 5D**). In addition, the diagnostic validity of DLBCL biomarkers was verified by ROC analysis, and an AUC greater than 0.6 was considered to have good specificity and sensitivity for DLBCL diagnosis. As shown in **Figure 5E**, the AUC values of CDC6, CDC20, KIF20A, STIL, and TOP2A were 0.799, 0.909, 0.783, 0.757, and 0.825, respectively. Finally, we performed a prognostic assessment of these hub genes in DLBCL and found that the expression of these genes had a significant impact on the prognosis of DLBCL patients. When the expression of CDC6, CDC20, KIF20A, STIL, and TOP2A was low, the prognosis of DLBCL patients was better (p < 0.05) (**Figure 5F**).

### Immunohistochemistry, genetic mutations, and clinical relevance of hub genes in DLBCL

**Figure 6:**
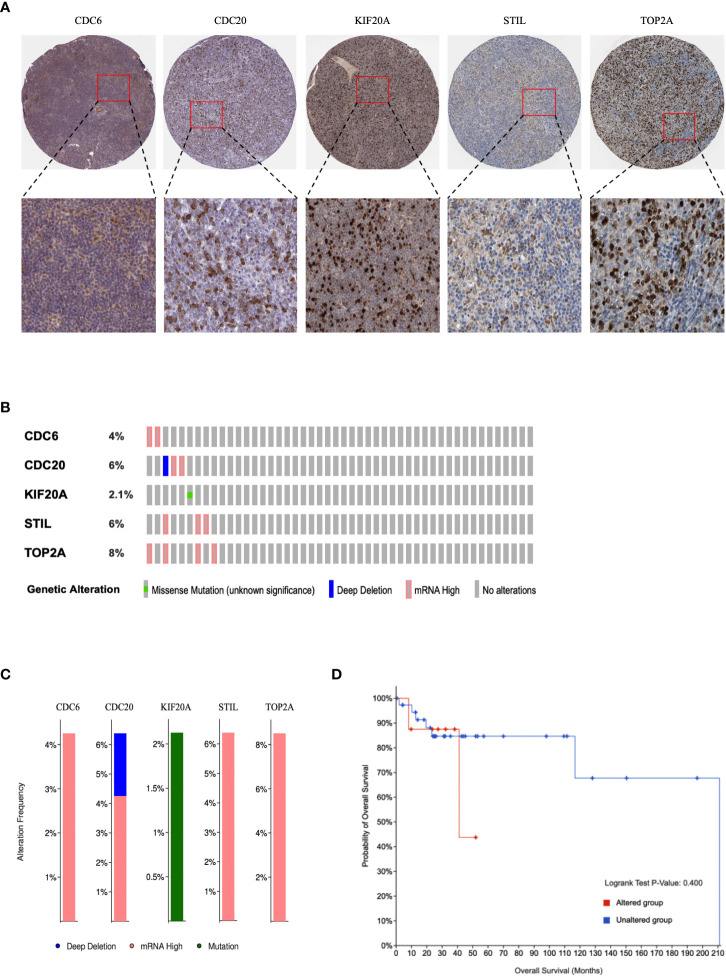
Immunohistochemical and genetic mutations analysis of hub genes in DLBCL. **(A)** Representative IHC images of hub genes with corresponding antibody in DLBCL. **(B)** Alteration summary as OncoPrint heatmap. **(C)** Distribution of genetic alteration frequencies. **(D)** Kaplan–Meier survival curves of overall and disease-free survival in DLBCL patients with altered and unaltered genes.

The HPA portal contains IHC images of hub genes in DLBCL samples. Overall, IHC samples of CDC6, CDC20, KIF20A, STIL, and TOP2A showed that the proteins were highly expressed in DLBCL (**Figure 6A**), like mRNA differential expression analysis. We queried hub genes in cBioPortal to discover their genetic alterations in DLBCL. Genetic alterations (also called genetic aberrations) refer to chromosomal abnormalities and genetic mutations, often the main drivers of cancer progression. Data from 48 DLBCL samples from Firehose Legacy were examined. They showed that CDC6, CDC20, KIF20A, STIL, and TOP2A were altered at frequencies of 4%, 6%, 2.1%, 6%, and 8%, respectively (**Figure 6B**). The frequency distribution of gene alterations is shown in **Figure 6C**. Subsequently, we defined DLBCL patients without genetic alterations as the “unchanged group” and the remaining patients as the “altered group.” Although survival analysis detected hub gene alterations with a P value greater than 0.05 for disease-free survival compared with unaltered DLBCL patients, based on trend analysis of the curves, we found that alterations in hub genes had a positive effect on the prognosis of DLBCL patients have potential adverse effects (**Figure 6D**).

### Diagnostic effectiveness of the biomarkers for DLBCL

**Figure 7:**
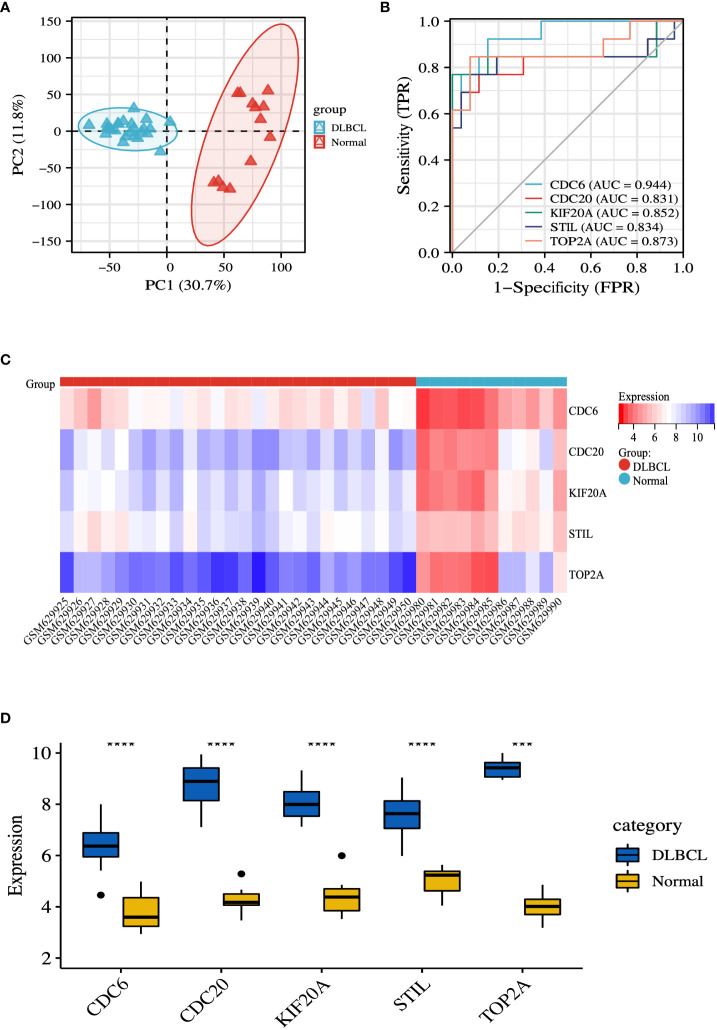
Validation of hub genes. **(A)** PCA diagram of samples based on expression abundance. **(B)** The GSE25638 dataset was used to validate the diagnostic effectiveness of the biomarkers by ROC analysis. **(C)** The GSE25638 dataset was used to validate the expression of hub genes, the results of which were presented as a heatmap. **(D)** Detailed expression of hub genes. ***P < 0.001; ****P < 0.0001.

To ensure more reliable results, we used the microarray dataset GSE25638 for validation. Based on the GSE25638 dataset, DLBCL was completely differentiated from the normal group by PCA analysis (**Figure 7A**). The diagnostic validity of the hub genes was verified by ROC analysis. The results suggest that in the GSE25638 dataset, the AUC values of CDC6, CDC20, KIF20A, STIL and TOP2A were 0.944, 0.831, 0.852, 0.834 and 0.873, respectively (**Figure 7B**). In addition, the expression levels of CDC6, CDC20, KIF20A, STIL, and TOP2A were shown in the heatmap (**Figure 7C**). Statistical analysis of differences between groups indicated that CDC6, CDC20, KIF20A, STIL, and TOP2A were all highly expressed in the DLBCL group (P < 0.001) (**Figure 7D**).

### Correlation analysis between growth regulatory pathways and hub genes in DLBCL

**Figure 8:**
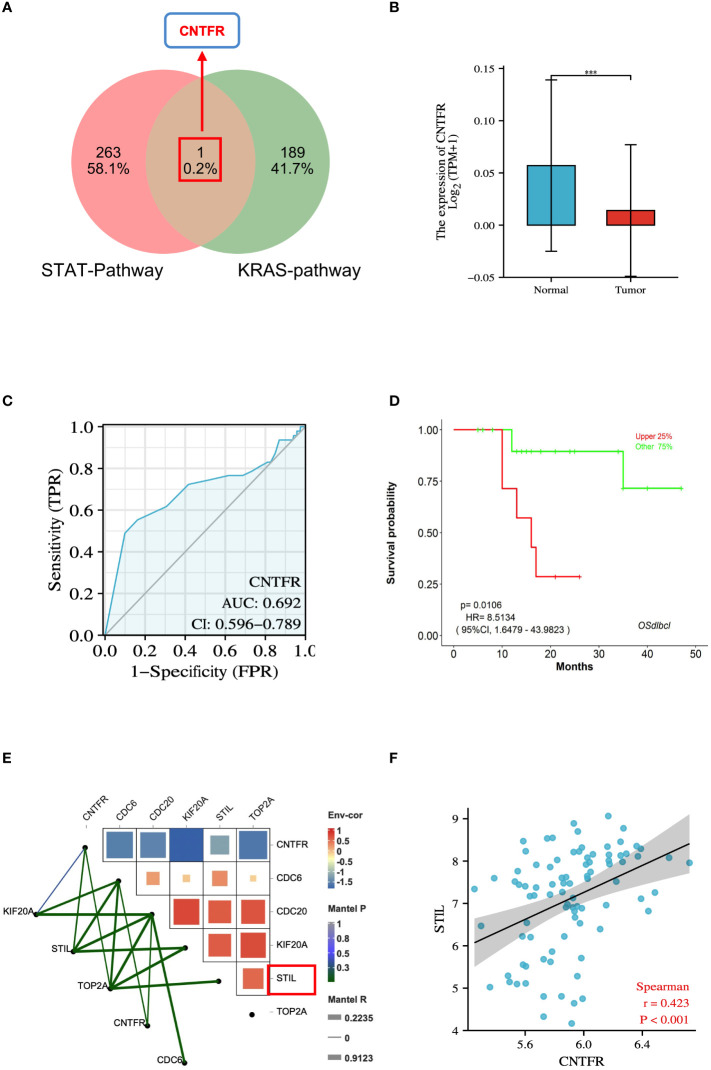
Integrative analysis of genes associated with growth regulatory pathways in DLBCL. **(A)** Venn diagram of 1 overlapped DEGs of STAT-pathway and KRAS-pathway. **(B)** Expression levels of the CNTFR between DLBCL and normal tissues in TCGA database. **(C)** ROC analysis of CNTFR. **(D)** Survival analysis of CNTFR. P < 0.05 was regarded as the critical point with statistical significance. **(E)** Correlation between hub genes and CNTFR in DLBCL. **(F)** Spearmen’s analysis on the correlation between STIL and CNTFR. ***P < 0.001.

To investigate the relationship between hub genes and tumor growth regulation, we used the venny tool to integrate genes related to STAT and KRAS pathways. We took their overlapping portion, identifying the overlapping gene CNTFR (**Figure 8A**). CNTFR was evaluated using the TCGA database. The results suggested that the mRNA expression of CNTFR in DLBCL was lower than that in the normal group (P < 0.001) (**Figure 8B**). The diagnostic validity of CNTFR was verified by ROC analysis. The results suggested that the AUC value of CNTFR was 0.692 (**Figure 8C**). The LOGpc database was used to evaluate the prognosis of genes. The results showed that when CNTFR was highly expressed in DLBCL, the survival rate of patients decreased (P < 0.05) (**Figure 8D**), which indicated that the changes in CNTFR had the potential adverse effects for the prognosis of DLBCL patients. In addition, we evaluated the correlation between hub genes and CNTFR, and the heatmap results of correlation analysis indicated that the expressions of CDC6, CDC20, KIF20A, STIL, TOP2A, and CNTFR were all correlated (P < 0.05), and the difference between STIL and CNTFR was significantly higher. The highest correlations were observed (**Figure 8E**). In addition, the results of spearman analysis further confirmed this result (**Figure 8F**).

### Experimental verification of hub genes

**Figure 9:**
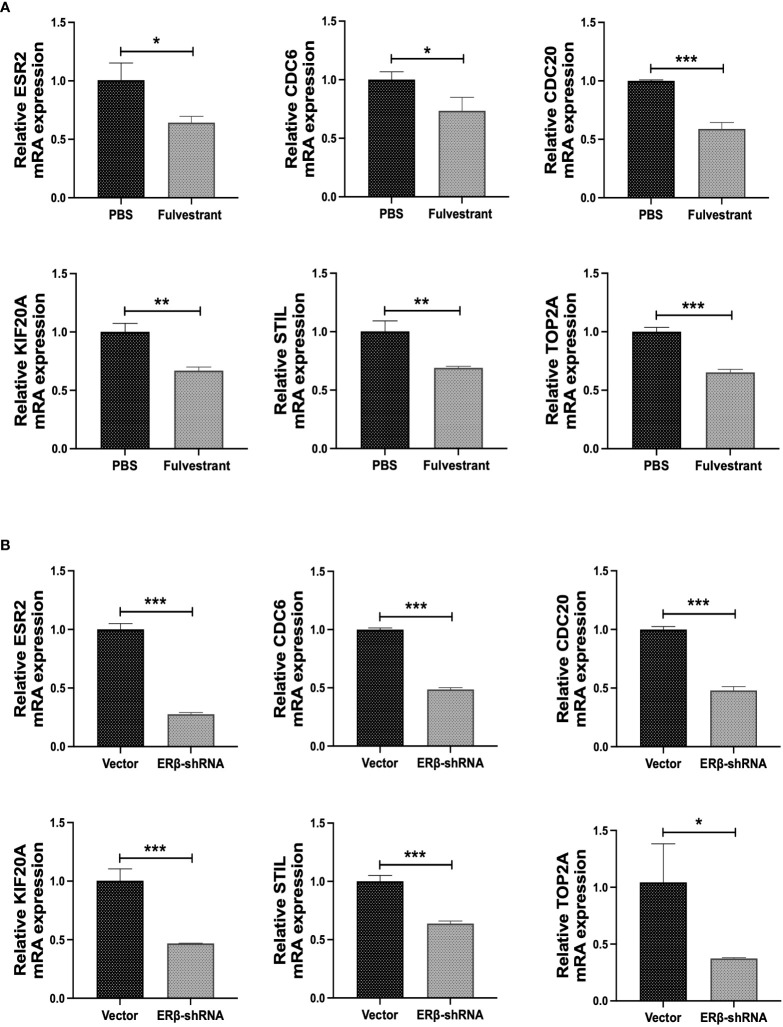
Experimental verification of hub genes. **(A)** The mRNA levels of ESR2, CDC6, CDC20, KIF20A, STIL and TOP2A were detected after cells were treated with 100nM fulvestrant. **(B)** The mRNA levels of ESR2, CDC6, CDC20, KIF20A, STIL and TOP2A were detected after ERβ-shRNA transfected cells. *P < 0.05; **P < 0.01; ***P < 0.001.

To further validate the results of the bioinformatics analysis, we designed ER inhibitors fulvestrant and ERβ-shRNA. BJAB Cells were treated with 100 nM fulvestrant and ERβ-shRNA transfection, respectively. Changes in the expression of hub genes at the mRNA level were detected. The results showed that the expression of CDC6, CDC20, KIF20A, STIL, and TOP2A was decreased at the mRNA level after BJAB cells were treated with fulvestrant (P < 0.05) (**Figure 9A**). In addition, after transfection of ERβ-shRNA into BJAB cells, the expressions of CDC6, CDC20, KIF20A, STIL, and TOP2A decreased at the mRNA level, which further verified the reliability of the results (P < 0.05) (**Figure 9B**). These data suggest that ER positively regulates CDC6, CDC20, KIF20A, STIL, and TOP2A expression in BJAB cells, which is consistent with the results predicted by bioinformatics analysis.

## Discussion

Currently, targeting DLBCL remains a major clinical challenge, with approximately 30% of patients unable to achieve clinical cure (23, 33). Understanding the molecular pathogenesis of DLBCL identifies druggable molecular pathways. New drugs for DLBCL are being studied and focusing on the optimal sequencing of emerging DLBCL drugs is necessary (34). DLBCL is a heterogeneous disease in terms of morphology, immunophenotype, and molecular features, and its pathogenesis reflects this (35–37). Studies have shown that the incidence of DLBCL in women is lower. Still, it increases after the age of 50, usually leading to menopause, suggesting that sex hormones may have a protective role for women in developing DLBCL (19, 20, 38). A previous study indicated that exposure to estrogen in oral contraceptives or postmenopausal hormone replacement during pregnancy reduces the risk of aggressive lymphoma (39). Females were an important favorable prognostic factor compared with males in NHL (23, 40). Yakimchuk K et al. showed that T-cell lymphomas grow faster in ovariectomized mice than in normal female mice (41). In addition, blocking estrogen synthesis by aromatase inhibitors can accelerate lymphoma progression (42). In ovariectomized mice, estradiol treatment slows lymphoma growth (26). Animal-level studies found that human DLBCL transplanted into female mice grew significantly slower than in male mice (43). These results suggest that sexually dimorphic mechanisms that influence tumorigenesis and progression in DLBCL are widespread, likely due to differences in physiological processes such as sex hormone signaling, especially estrogen.

To date, no functional test is capable of screening, and patients must be managed effectively once they are diagnosed. Therefore, identifying the characteristics and regulatory pathways of unique estrogen receptor genes relevant to their pathogenesis and prognosis is of interest. Here, we examined the gene expression profiling of GSE56315 to discover dysregulated hub genes and pathways to understand DLBCL pathogenesis further and provide potential biomarkers.

First, based on differential gene expression analysis and GSEA enrichment analysis, we identified two crucial signaling pathway modules (estrogen receptor signaling and growth regulation signaling pathways) involved in DLBCL regulation. The estrogen signaling receptor pathway includes estrogen receptor early and estrogen receptor late. As we have analyzed above, estrogen receptor signaling, the biological rhythm signal of tumors, plays an essential regulatory role in the occurrence and development of DLBCL. Growth regulation pathways mainly include KRAS signaling, IL6 Jak STAT3 signaling, and IL2 STAT5 signaling. Studies have shown that the Ras signaling pathway contributes to the survival of human T-cell leukemia/lymphoma virus type 1 (HTLV-1) tax-positive T cells (44). Tiacci E et al. document the functional role of mutant STAT6 in maintaining tumor cell viability and show that genetic and functional mutations synergize with disruption of JAK-STAT pathway inhibitors to promote the growth of classical Hodgkin lymphoma (45). Viganò E et al. showed that somatic IL4R mutations in primary mediastinal large B-cell lymphoma led to activation of JAK-STAT signaling (46). In addition, some studies have found that ritotinib inhibits the proliferation of Hodgkin lymphoma cells and induces apoptosis by inhibiting the JAK/STAT signaling pathway (47). The above results suggest that the estrogen receptor signaling pathway plays a vital role in regulating the growth of DLBCL and the activation and regulation of tumor growth signals dominated by KRAS and JAK/STAT signals in DLBCL.

By integrating bioinformatics analysis, gene expression levels and survival analysis, we identified five differentially co-expressed hub genes associated with estrogen receptor signaling associated with DLBCL pathogenesis. These genes include CDC6, CDC20, KIF20A, STIL, and TOP2A. Construction of ROC curves yielded higher AUC values, indicating that these genes can accurately distinguish non-tumor control and DLBCL groups and may be potential biomarkers. We performed a prognostic assessment of these hub genes in DLBCL and found that the expression of these genes had a significant impact on the prognosis of DLBCL patients. The IHC experimental data obtained from the HPA database showed that the hub genes were highly expressed in DLBCL tissues. In addition, genetic alteration analysis suggests that these genes are altered in DLBCL, and the prognosis of DLBCL patients in the genetic alteration group is poor, which also provides a valuable basis for medical research.

There are few experimental studies on the role of hub genes in DLBCL. CDC6 has been reported to be associated with immune infiltration and is a potential prognostic biomarker in glioma (48). Jasmonic acid induces biological activity in Arabidopsis and selectively inhibits breast cancer cell growth *via* CDC6 and mTOR (49). CDC20 is a novel biomarker that improves the clinical prediction of epithelial ovarian cancer (50). Increased CDC20 expression correlates with differentiation and progression of pancreatic duct adenocarcinoma (51). KIF20A, a kinesin, promotes the progression of castration-resistant prostate cancer through autocrine activation of the androgen receptor (52). In liver cancer, low expression of KIF20A inhibits cell proliferation, promotes chemosensitivity, and is associated with a better prognosis (53). STIL acts as an oncogene driver in a primary cilia-dependent manner in human cancer (54). STIL is integral in regulating cancer cell motility through plate oval accumulation of the ARHGEF7-PAK1 complex (55). TOP2A can be used as an indicator for early detection and diagnosis of aggressive prostate cancer subsets (56). Alterations in the TOP2A gene are associated with anthracycline adjuvant sensitivity in human breast cancer (57). A related study on lymphoma indicated that the CDC6 G1321A polymorphism was associated with a reduced risk of non-Hodgkin lymphoma (58). MDM2-P53 signaling-mediated upregulation of CDC20 promotes the progression of human diffuse large B-cell lymphoma (59). Expression of nm23, TOP2A, and VEGF are potential prognostic biological factors in peripheral T-cell lymphoma (60).

To investigate the validity of these biomarkers in diagnosing DLBCL, we performed validation using the microarray dataset GSE25638. Consistent with the above analysis results, the expressions of CDC6, CDC20, KIF20A, STIL, and TOP2A in DLBCL cells were significantly elevated and statistically different. The diagnostic validity of DLBCL biomarkers was validated by ROC analysis, and the results clearly showed that CDC6, CDC20, KIF20A, STIL, and TOP2A were specific and sensitive in diagnosing DLBCL. In addition, we further integrated the genes related to the growth signaling pathway and identified the key target CNTFR. The potential value of the target was validated through a series of expression analyses, ROC, and survival analyses. Correlation analysis found that five effective biomarkers (CDC6, CDC20, KIF20A, STIL, and TOP2A) were associated with CNTFR, and STIL had the strongest correlation with CNTFR. Spearman’s analysis indicated that R=0.423 and P<0.001. This also provides essential information that STIL, as an estrogen receptor signaling-related gene, in DLBCL, may regulate growth signals by targeting CNTFR and play a role in regulating tumor growth and development.

To confirm the value of hub genes as DLBCL biomarkers, we performed *in vitro* experiments. After BJAB cells were treated with the ER inhibitor fulvestrant, the expression of hub genes was detected by RT-qPCR experiments. The results suggested that the mRNA expression levels of CDC6, CDC20, KIF20A, STIL, and TOP2A decreased, consistent with the analysis results. To further confirm the reliability of the results, we transfected BJAB cells with shRNA plasmid to knock down ER. The results also suggested that the mRNA expression levels of CDC6, CDC20, KIF20A, STIL, and TOP2A were reduced. As we know, BJAB is an EBV-negative Burkitt-like lymphoma cell line. Based on the laboratory conditions, as Burkitt and DLBCL are close because of the germinal center “origin,” we chose the BJAB cells used for target validation. Our experiment results effectively indicate these targets may be universal in lymphoma (more evidence of lymphoma cell lines is needed). It also provides some good information for further research. In future studies, we will supplement corresponding lymphoma cell lines to ensure more rigorous research results. There are few reports on the regulatory relationship between CDC6, CDC20, KIF20A, STIL, TOP2A, and ER. Sparano JA et al. pointed out that TOP2A expression provides prognostic information in ER-positive breast cancer (61). Rody A et al. found that topoisomerase II alpha (TOP2A) gene expression has a high prophetic role in ER-positive breast cancer (62). In our study, the database-based prediction and mRNA level validation essentially highlighted the roles of CDC6, CDC20, KIF20A, STIL, and TOP2A. These hub genes are expected to become new target genes for ER.

TP53 mutation is considered a significant predictor of chemotherapy-refractory in DLBCL patients. Studies have shown that TP53 mutations are associated with poor prognosis in patients with DLBCL (63–65). Chiappella et al. also noted that in young patients with intermediate- and high-risk DLBCL, TP53 disruption due to genetic mutations identified a subgroup of patients with an abysmal prognosis. DLBCL cases with TP53 mutations have lower response rates to chemoimmunotherapy and lower survival rates for FFS and OS compared with patients with TP53 WT (66). Currently, there is a lack of effective treatment for patients with TP53 mutations in clinical practice, and the disease development can be appropriately delayed through targeted drug therapy. Still, the problem is that targeted drug resistance is widespread, and it is necessary to find new specific targeted drugs. Therefore, in our study, based on these predictions about new target genes for ER, it is desirable to develop investigational small molecules for DLBCL (e.g., novel monoclonals), which also provides new ideas for the treatment of clinical DLBCL patients.

DLBCL is a molecularly heterogeneous disease characterized by distinct sets of genetic and epigenetic alterations involving multiple functional signaling pathways regulated by genetic events, resulting in alterations at the transcriptional or translational level (67–71). Therefore, factors such as methylation, microRNA, and lncRNA should also be considered. This is a flaw in the specific conclusion of this study that changes in these mRNAs lead to a switch in signaling pathways. Another limitation of this study is the lack of validation experiments, which will be performed in our future work.

## Data availability statement

The original contributions presented in the study are included in the article/**Supplementary Material**. Further inquiries can be directed to the corresponding author.

## Author contributions

BC contributed to the study design, collection and interpretation of the data, the writing of the manuscript. TM, XQ, WZ, and NW contributed to the study design, and interpretation of the data. JL contributed to the study design, interpretation of the data, the writing of the manuscript, and the submission of the manuscript for publication. All authors contributed to the article and approved the submitted version.

## Funding

We thank the support from the National Key Research and Development Program of China (2021YFE0108000).

## Conflict of interest

The authors declare that the research was conducted in the absence of any commercial or financial relationships that could be construed as a potential conflict of interest.

## Publisher’s note

All claims expressed in this article are solely those of the authors and do not necessarily represent those of their affiliated organizations, or those of the publisher, the editors and the reviewers. Any product that may be evaluated in this article, or claim that may be made by its manufacturer, is not guaranteed or endorsed by the publisher.
