# Supplementary material for: Role of estrogen receptor signaling pathway-related genes in diffuse large B-cell lymphoma and identification of key targets via integrated bioinformatics analysis and experimental validation
Source: Front Oncol. 2022 Nov 29;12:1029998. doi: 10.3389/fonc.2022.1029998 (PMC9749266; doi:10.3389/fonc.2022.1029998)
Supplement: Supplementary file 1 [file DataSheet_1.docx]

**Materials and Methods**

**Principal Component Analysis (PCA)**

Pearson's correlation test verified the reproducibility of data within each group. The within-group data repeatability of the dataset was tested by sample cluster analysis. Statistical analysis was performed using the R language, and the "ggplot2" package presented the results.

**GO and KEGG enrichment analysis**

Venn diagrams were constructed using the online tool Venny (v 2.1, https://bioinfogp.cnb.csic.es/tools/venny/) to identify overlapping modules and genes in the dataset. DAVID (http://david.abcc.ncifcrf.gov/) is an annotation, visualization, and comprehensive discovery database, an online tool for gene function classification, gene analysis, and assessment of the biological function of genes. Here, we performed GO and KEGG enrichment analysis using the DAVID database to investigate the role of DEGs. P < 0.05 was regarded as the critical point with statistical significance.
